# Single-cell RNAseq for the study of isoforms—how is that possible?

**DOI:** 10.1186/s13059-018-1496-z

**Published:** 2018-08-10

**Authors:** Ángeles Arzalluz-Luque, Ana Conesa

**Affiliations:** 1 Genomics of Gene Expression Laboratory, Centro de Investigación Principe Felipe (CIPF), 46012 Valencia, Spain; 2 Department of Microbiology and Cell Science, Institute for Food and Agricultural Sciences, Genetics Institute, University of Florida, Gainesville, Florida, 32611 USA

## Abstract

Single-cell RNAseq and alternative splicing studies have recently become two of the most prominent applications of RNAseq. However, the combination of both is still challenging, and few research efforts have been dedicated to the intersection between them. Cell-level insight on isoform expression is required to fully understand the biology of alternative splicing, but it is still an open question to what extent isoform expression analysis at the single-cell level is actually feasible. Here, we establish a set of four conditions that are required for a successful single-cell-level isoform study and evaluate how these conditions are met by these technologies in published research.

## Introduction

Sequencing technologies have had a profound impact on the way we conduct transcriptome research, enabling access to the entire span of transcripts in a biological sample thanks to RNAseq. RNAseq applications range from classic evaluations of differential transcript or gene expression between samples [1] to more-diverse problems such as the characterization of gene expression dynamics [2], gene boundaries [3, 4], translation efficiency [5] or RNA–protein interactions [6, 7], to name a few. In the past few years, two RNAseq applications have raised particular interest for describing the complexity and diversity of transcriptional regulation—single-cell RNAseq [8] and the study of alternative splicing on a large scale [9, 10]. Bulk RNAseq experiments average gene expression across populations of cells and thus preclude capture of cell-to-cell variability. This motivated the development of a single-cell strategy for RNAseq [8], and efforts have been relentless to improve the strategy ever since. To this date, single-cell RNAseq has provided valuable insight into cell differentiation [11–15], complex tissue and rare cell population composition [16–19] or tumor heterogeneity [20, 21] and growth [22], and it constitutes a cutting-edge technology in biological research. As for the field of isoform transcriptomics, early studies showed high levels of tissue-specific and developmentally regulated alternative splicing (AS) events [9, 10, 23–25], which was interpreted as an extra layer of phenotypic complexity. Since then, RNAseq has served to characterise an increasing number of AS events with well-established roles in biological processes, namely cell proliferation and survival, differentiation, homeostasis, responses to stress and, when altered, disease. These events and their mechanisms of regulation have been thoroughly reviewed over the past few years [23, 26–31], setting the notion of alternative splicing as a complex, tightly regulated, functionally relevant process, although still poorly understood on a global scale. Moreover, there is an ongoing controversy surrounding their biological relevance [32–34].

In contrast to the high abundance of both single-cell RNAseq and bulk-level alternative splicing studies, cases where single-cell transcriptome profiling is used to address the variability of isoforms are scarce (Table 1). However, quite contrarily to what might be suggested by the extant gap in the literature, daring to go beyond the bulk is essential to answer some of the questions concerning the expression patterns of alternative isoforms. The recently found heterogeneity in isoform expression mechanisms in single cells [35–38] is highly intriguing to the scientific community, and raises the question of whether this diverse and complex isoform expression landscape constitutes an additional layer of gene expression regulation or is solely a result of the stochastic functioning of the alternative splicing machinery. There is currently no doubt that single-cell isoform studies could be the key to resolve this fundamental problem.

**Table 1:** Comparison of published single-cell RNAseq isoform studies

		Reference	Main focus of the study	Full-length isoforms?	Computatio-nal method	Aim	Organism, cell type	Library prep	Feature or event targeted
Illumina sequencing		Ramskold et al. [39]	Single-cell RNAseq, genes	✗	MISO *Developed for bulk RNAseq*	Experimental protocol development *Library preparation*	Human, cancer cells	Smart-seq	Exon inclusion quantification
		Shalek et al. [36]	Single-cell RNAseq, genes and isoforms	✗	MISO *Developed for bulk RNAseq*	Single-cell heterogeneity in immune response	Mouse, BMDCs	Smart-seq	Exon inclusion quantification
		Zhang et al. [40] *Data from Shalek* et al. [36]	Bulk RNA-seq, isoforms	✗	WemIQ *Developed for bulk RNAseq + single-cell validation*	Computational method development *Isoform identification*	Mouse, BMCDs	Smart-seq	Single-cell bias in differential isoform detection
		Marinov et al. [35]	Single-cell RNAseq, genes and isoforms	✗	Pervouchine et al. [48] *Developed for bulk RNAseq*	Single-cell isoform and gene expression heterogeneity	Mouse, lymphobl-astoid cells	Smart-seq	Novel splice junctions, exon inclusion quantification
		Velten et al. [44]	Single-cell RNAseq, isoforms	✗	BATBayes	3′ UTR variability among genes and cells	Mouse, ESCs	BATSeq	Alternative poly(A) sites
		Welch et al. [42] *Data from Buettner* et al. [17]	Single-cell RNAseq, isoforms	✗	SingleSplice	Computational method development *Differential isoform usage*	Mouse, ESCs	Smart-seq/C1	Differential isoform usage
		Karlsson et al. [45] *Data from Zeisel* et al. [18]	Single-cell RNAseq, isoforms	✗	Alignment to FANTOM 5 database *Developed for CAGE*	Single-cell isoform expression heterogeneity	Mouse, brain cells	STRT-seq/C1	Alternative TSS
		Song et al. [38]	Single-cell RNAseq, isoforms	✗	Expedition	Computational method development *Differential exon inclusion/exclusion*	Human, iPSCs, NPCs and MNs	Smart-seq/C1	Exon inclusion quantification
		Huang et al. [43] *Data from Wu* et al. [49] *and Scialdone* et al. [50]	Single-cell RNAseq, isoforms	✗	BRIE	Computational method development *Differential exon inclusion/exclusion*	Human HCT116 cells + mESCs	Smart-seq + Smart-seq2	Exon inclusion quantification
Single-molecule sequencing	Oxford Nanopore	Byrne et al. [46]	Single-cell RNAseq, isoforms	✓	Mandalorion	Computational method development *Isoform structure and quantification*	Mouse, B1 cells	Smart-seq2	TSS, TTS, exon inclusion, intron retention, alt. 3′ and 5′ splice sites
	PacBio	Karlsson and Linnarsson [47]	Single-cell RNAseq, isoforms	✓	Self-designed pipeline	Single-cell isoform expression heterogeneity	Mouse, oligoden-drocytes and VLMCs	STRT-seq/C1	TSS, TTS, exon inclusion, alt. 3′ and 5′ splice sites

Illumina involves short-read sequencing, and single-molecule sequencing involves long-read technologies. Studies are classified per ‘focus’, either bulk-RNAseq, single-cell RNAseq for gene expression or isoform single-cell RNAseq (or both). Only ‘computational methods’ used for isoform identification/quantification are specified. ‘Full-length’ is only considered as such when isoforms were reconstructed end-to-end, regardless of whether library preparation was full-length or not. Text in *italics* adds complementary information on the aim of the computational method/library protocol developed. When specified, the study was performed on data generated by other authors. ‘Feature/event targets’ refer to the approach taken to study isoform diversity, or to a specific aspect of it that is tackled. For more information, readers should refer to this review’s analysis or to the referenced papers

*BMDC* bone-marrow-derived dendritic cell, *ESC* embryonic stem cell, *iPSC* induced pluripotent stem cell, *mESC* murine embryonic stem cell, *MN* motor neuron, *NPC* neural progenitor cell, *TSS* transcription start site, *TTS* transcription termination site, *UTR* untranslated region, *VLMC* vascular and leptomeningeal cell

Transcriptome-level analyses of isoforms have been performed as a part of single-cell RNAseq gene expression publications [35, 39] or in bulk studies of isoform diversity [40], but merely as a proof-of-concept. Usually, the aim of these studies was never to address single-cell isoform diversity, but to test the performance of the experimental protocols or computational tools in this scenario. In such a limited frame, the former studies accomplished identification of only a small number of above-noise splicing differences among single cells and lacked in-depth evaluation of results. For some years, only methods developed for RNAseq, mainly ‘mixture of isoforms’ (MISO) [41], were used in single-cell isoform research [35, 36], and it was not until recently that computational strategies tailored to the particularities of single-cell RNAseq began to appear [38, 42, 43]. Notably, the use of short-read sequencing and the unavailability of tools for comprehensive isoform structure analysis have limited most research to solely quantification of exon inclusion levels [35, 36, 38, 39, 43] or targeting specific regions of the transcripts—that is, the 3′ untranslated region (UTR) for alternative polyadenylation sites [44] or the 5′ UTR for transcription start sites (TSSs) [45]. Recent studies applying single-molecule sequencing technologies, however, have succeeded in characterizing full-isoform structures [46, 47] on a limited number of cells (four to six), incorporating the entire span of alternative splicing events. Of note, most of the above-cited studies make use of publicly available computational methods (i.e. [41, 48]) and datasets (i.e. [17, 18, 36, 49, 50]) (Table 1).

**Fig. 1:**
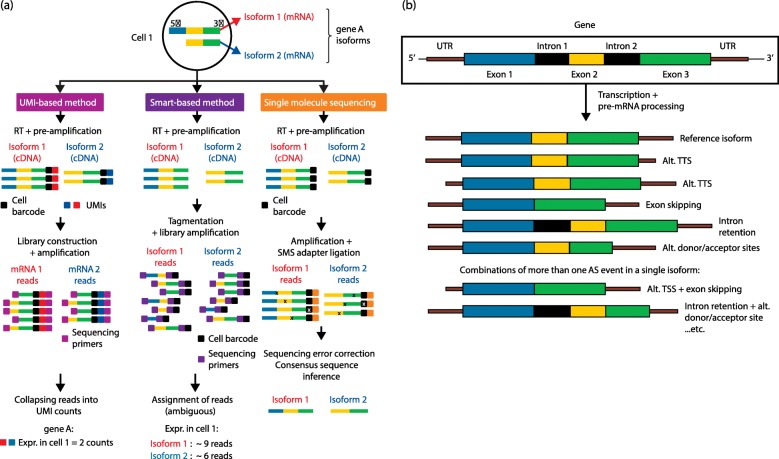
Single-cell mRNA sequencing methods and sources of mRNA variation. **a** Methodological approaches to single-cell isoform studies. The combination of library preparation and sequencing technologies yields three distinct methods to capture isoform diversity. UMI-based methods are limited to sequencing of the 3′ (or 5′ end), which enables usage of UMIs to capture efficiently PCR bias in addition to early cell barcoding, even if they are particularly suited to quantify expression at the gene level. Smart-based methods produce short reads across the entire transcript length, although they require late cell barcoding (barcodes inserted in tagmentation), cannot accommodate UMIs, and the reads might be difficult to assign unambiguously to an isoform. Single-molecule sequencing allows sequencing of each transcript molecule in a single read and provides full isoform connectivity, although it suffers from a high prevalence of sequencing errors. **b** Sources of transcript variation that yield alternative isoforms and their position along the transcript. When compared with a reference isoform (for convenience, that including all exons, no introns and the complete UTRs), alternative TSSs (transcription start sites) and TTSs (transcription termination sites) are generated during the transcription process by shortening of the UTRs. Processing of the pre-mRNA eliminates or retains introns and exons, adding variability to the isoforms that can be generated from the gene. In addition, more than one event can simultaneously be present in the same isoform, and consequently isoform diversity will increase with the number of possible combinations of AS events. *Alt.* alternative, *RT* reverse transcription, *UMI* unique molecular identifier

In the framework of single-cell isoform studies, three combinations of library preparation and sequencing technologies are available for data generation (Fig. 1a):

- Within short-read technologies (Illumina), two methods can be distinguished depending on the library preparation strategy of choice:
  - ○ UMI-based methods provide short reads from a fragment of the 3′ or 5′ end and include a unique molecular identifier (UMI) as a means of accounting for amplification bias.
  - ○ Smart-based methods provide short reads spanning the entire length of the transcript but cannot accommodate UMIs for a more accurate expression quantification.
- Long-read technologies (a.k.a. single-molecule sequencing), by contrast, capture an entire transcript molecule in a single sequencing read.

Here, we focus on outlining the limitations of these three methodological approaches in their application to AS and isoform expression. To this end, we first establish an ideal set of requirements for a successful isoform study and evaluate to what extent they are fulfilled in single-cell RNAseq publications (Table 1). Based on the knowledge drawn from this analysis, we show a simple computational simulation that reveals the limiting factors intrinsic to each of the three methods in a real experimental setting. Finally, we discuss the biological questions that single-cell isoform studies could address and the future perspectives of the approach.

## Ideal conditions for single-cell isoform RNAseq

**Fig. 2:**
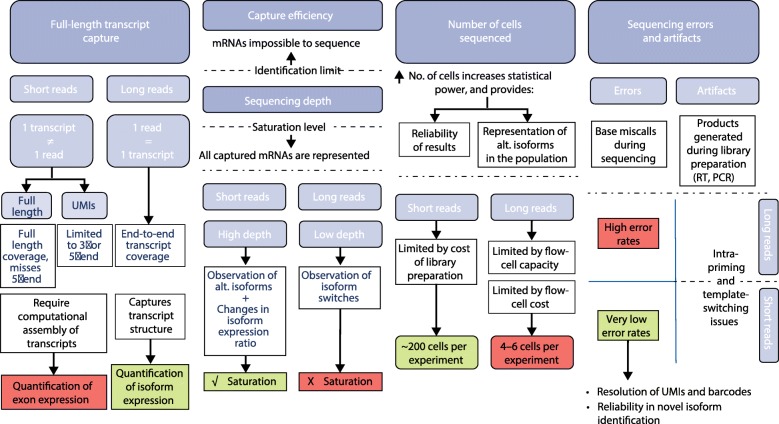
Summary of limitations of the four ideal conditions for successful studies of single-cell RNAseq isoforms. From *left* to *right*, the importance and current limitations of full-length transcript sequencing, capture efficiency and sequencing depth, the number of cells sequenced, and sequencing errors and artefacts for isoform detection are presented in the diagram. Each is discussed in the main text. *Alt.* alternative, *RT* reverse transcription, *UMI* unique molecular identifier

Analysing isoforms at the single-cell level requires a deep understanding of the biology of alternative splicing, regarding both structural complexity and the nature of changes in their expression level. Isoform diversity is determined by the number of exons, introns, the TSS and transcription termination site (TTS) and alternative donor/acceptor sites that are contained in a gene, but more importantly by the different combinations of them that are expressed as transcripts (Fig. 1b). Hence, each AS event is very likely to be present in several different isoforms. To add to this complexity, isoforms within a gene are very differently expressed, typically showing, for a particular cell type, a dominant (i.e. very highly expressed) isoform and several others with significantly lower expression values. In the light of these two particularities—the constraints and the biases intrinsic to single-cell RNAseq—we discuss a number of parameters important for single-cell RNAseq isoform analyses (Fig. 2).

### Full-length transcript capture

Different types of splicing variations can occur at different points of the mRNA molecule (Fig. 1b). Hence, partial sequencing of the transcript will naturally overlook a fraction of the events and might make it impossible to distinguish some of the isoforms of a gene. Such is the case in library preparation protocols for Illumina sequencing that include unique molecular identifiers (UMIs) [51] UMIs are short, random oligonucleotides that are incorporated into cDNA before PCR, in the reverse-transcription step, and are designed so that, by probability, cDNA molecules belonging to the same gene will have a different UMI. This system allows for molecular counting after PCR. Thus, non-linear amplification, which hinders accurate expression quantification, can be corrected by collapsing reads with matching UMIs and mapping sites. This is especially relevant to single-cell RNAseq owing to the extra PCR cycles necessary to obtain enough cDNA for sequencing, which typically add up to between 30 and 40 cycles and introduce a non-trivial amount of bias. However, UMI-based methods provide reads coming only from the 3′ end of transcripts, where the UMI is attached in most current protocols (CEL-seq2 [52], inDrop [53], Drop-seq [54], MARS-seq [55] or SCRB-seq [56]). Among these, STRT-seq [51] constitutes an exception, since the UMI is attached at the 5′ end. UMI methods therefore enable sequencing of only a fragment of the transcript, preventing isoform discrimination when differences are not located in this part of the sequence.

As a result, a trade-off is established, by which isoform studies therefore necessitate relinquishing UMI usage in favour of strategies that provide full-length transcript information. The Smart-seq [39] and Smart-seq2 [57] protocols accomplish this by including an enhanced reverse transcription (RT) step that ensures capture of the entire transcript and full-length cDNA synthesis, and hence are particularly suitable for isoform studies. The resulting system, known as SMART [58], uses the Moloney murine leukemia virus (MMLV) reverse transcriptase to leave a 5′ oligonucleotide overhang after the enzyme has reached the end of the first strand, which is then used for template-switching (i.e. priming and synthesis of the second strand of the cDNA).

Single-molecule sequencing (SMS) technologies are an alternative to Illumina for sequencing SMART-generated libraries. Illumina’s tagmentation generates many short reads from the same transcript, which requires subsequent assembly of the transcriptome by computational methods. Assembly tools fail to recover the structure of the different isoforms and limit quantification to the level of exon expression. Alternatively, sequencing the full transcript in one read would facilitate isoform identification without the need for an assembly step. Current technologies that enable this are single-molecule real-time (SMRT) sequencing, by Pacific Biosciences (known as PacBio), and Oxford Nanopore Technologies’ MinION portable sequencer. Although different (for instance, Oxford Nanopore allows direct RNA sequencing, whereas cDNA synthesis is essential when sequencing with PacBio), both platforms have in common that the output reads are several kilobases long. Hence, as a rule, one read equals one transcript for both Oxford Nanopore and PacBio data, which makes them an attractive alternative for isoform studies.

### Sequencing depth

The low amount of starting material in single cells hinders capture efficiency and causes the appearance of transcript ‘drop-outs’—that is, the identification of a gene as unexpressed owing to absence of transcripts during reverse transcription [35, 59]. This mostly affects genes that are expressed at very low levels [60], for which zero-expression values cannot be distinguished from true, biological absence of expression. Therefore, mRNA capture efficiency sets a limit to the total number of transcripts that can be detected in single-cell RNAseq, but, for transcripts expressed above this detection limit, sequencing depth (coverage) is the key to maximize sensitivity (i.e. the probability of capturing a particular transcript in the cell [61]). The general consensus in the field concerning the level of depth at which saturation is achieved is that sequencing beyond one million Illumina reads per cell barely adds any new information [62, 63].

However, isoform expression requires different considerations concerning capture and depth. First, isoforms are more sensitive to high drop-out rates. As alternative (i.e. non-dominant) isoforms are typically expressed at low levels, the probability of missing them is high, and thus isoform diversity per gene can easily be underestimated. In addition, the saturation limit of single-cell RNAseq has been set regarding library complexity at the gene level, and thus reaching saturation at the isoform level could potentially require more than one million reads per cell.

In the context of isoform expression, lower depth might suffice when changes in isoform expression are *isoform switches*—that is, changes in the more highly expressed isoform. In this case, as long as the sequencing is deep enough to observe the most highly expressed isoform, cell-to-cell differences in splicing for a given gene will be detected. However, isoform expression often comprises modifications in the ratio of expression of the gene isoforms, which will only be detected when depth goes beyond the expression levels of both isoforms.

On a general note, the quality filtering steps required in single-cell RNAseq also apply and will have an impact on isoform studies, even though they are not specific to them. They comprise, first, removal of low-quality cells (for instance, cells where a low number of features are detected [64]) and, second, filtering of features that have zero expression in a high proportion of the cells. Notably, a stringent filtering of features is likely to decrease the number of alternative isoforms detected, which are expressed poorly and/or unevenly captured across cells.

### Number of cells sequenced

Sequencing a high number of cells contributes favorably to the power of an analysis—that is, the ability to characterize with high confidence the expression patterns among cells in the population [61]. Sequencing large sets of cells can therefore yield significant advances in our understanding of isoform expression at the single-cell level.

UMI-based library preparation protocols for Illumina currently enable processing of thousands of cells thanks to microfluidic implementations in droplet-based systems. Examples include inDrop [53], Drop-seq [54] or the 10× Genomics version of the inDrop protocol, which have raised the bar up to 250,000 cells [65]. However, these methods are not only restricted to the 3′ end but also provide low sequencing depth, which results in detection of fewer genes, and isoforms, per cell.

The Smart-based alternatives [39, 57] warrant high sensitivity (i.e. detection of up to 20,000 genes [61]) but are limited owing to cost and the necessity to prepare libraries manually for each cell. To reduce labour, the Smart strategy can be implemented using the Fluidigm C1 instrument for parallelization and automation of the library preparation process, although the system is still limited in terms of the cost per cell. By way of illustration, most recent studies show data from only 100 to 200 cells [38, 42] (Table 2).

**Table 2:** Summary of number of cells sequenced in studies of single-cell isoforms (short reads)

Reference	Ramsköld et al. [39]	Shalek et al. [36]	Marinov et al. [35]	Velten et al. [44]	Welch et al. [42]	Karlsson et al. [45]	Song et al. [38]
Reference for data	–	–	–	–	Buettner et al. [17]	Zeisel et al. [18]	–
Total number of cells	12	18	15	144	96	2816	206
Library preparation method	Smart-seq	Smart-seq	Smart-seq	BATSeq	Fluidigm C1/Smart-seq	Fluidigm C1/STRT-seq	Fluidigm C1/Smart-seq

In contrast, long-read technologies allow sequencing of very few cells [46, 47]. This constraint is intrinsic to the design of PacBio and Oxford Nanopore technologies, each of which is based on the use of flow-cells (that is, microfluidic chips containing the necessary structures for sequencing) capable of yielding a limited amount of total sequencing reads per run. Hence, cell multiplexing inevitably means limiting the number of reads that will be obtained per cell. Although trivial for a bulk population (as only a few samples will be sequenced in each flow-cell), this currently limits the number of cells per flow-cell that can be deep-sequenced to four to six [46, 47].

### Sequencing errors and artefacts

Sequencing errors are generated owing to base miscalls during sequencing, whereas artefacts usually appear during the amplification and reverse-transcription processes and comprise products that were not originally present in the original cell lysate [66]. These issues can have a significant impact in studies of single-cell isoforms.

Sequencing errors are highly frequent in long-read technologies, as sequencing is based on single molecules. Note that error rates in SMS refer to the consensus sequence and not the raw reads. PacBio implements a circular consensus sequencing (CCS) system, by which a cDNA molecule is sequenced in a circular manner, generating concatenated copies that are then collapsed in a consensus sequence where random errors are cancelled out. The accuracy of the final transcript sequence therefore depends on the number of copies present in the long read. Oxford Nanopore, by contrast, is equipped with a system based on the sequencing of the forward and reverse strands linked by a hairpin adapter, which are known as two-dimension (2D) reads. As a consequence, typical error rates for consensus PacBio sequences are in the range of ~ 2–5% [66], whereas, for Oxford Nanopore, the values go up to ~ 7% [67, 68]. This contrasts with the high accuracy of Illumina sequencing (~ 0.005% error rate).

Such high error rates are problematic for single-cell RNAseq isoform studies. A first constraint is the resolution of cell-specific barcodes (de-multiplexing) and UMIs. Given that single-cell RNAseq relies heavily on multiplexing strategies (to minimize batch effects and manual sample processing) as well as on UMI counts (to eliminate amplification bias), the occurrence of errors in these regions can add an extra challenge to analysis pipelines. Additionally, sequencing errors can lead to the erroneous identification of novel isoforms by introducing false alternative donor or acceptor sites. These errors frequently result in non-canonical splicing sites [66, 69] and might be corrected in downstream analyses.

Sequencing errors in SMS can be corrected using three different strategies: (a) a consensus of the long reads (discussed above); (b) clustering of reads belonging to the same transcript; and (c) complementary short-read sequencing, combining the accuracy of Illumina with the scaffolding potential of long reads. Sadly, compatibility with single-cell-level studies is only ensured in (a) as clustering requires high sequencing depth (not always achieved in single-cell sequencing) and complementary short-read sequencing is impracticable (the same cell cannot be sequenced using two different technologies). Some errors can therefore survive computational correction and result in erroneous mapping, leading to the discovery of false novel junctions.

Reverse transcription (RT) artefacts are also of high relevance to long-read-isoform studies. First, intra-priming events in genes with internal poly(A) sequences [70] can generate shorter cDNA artefacts that can be mistaken for isoforms with an upstream TTS. Additionally, mRNA molecules form secondary structures that can prevent access of the reverse transcriptase to certain fragments of the sequence, favouring template switching and skipping of these segments, which will appear as alternatively spliced isoforms [71]. SMS technologies have been shown to accumulate this kind of RT artefact and, in combination with sequencing errors, yield false new isoforms as a consequence. A software tool, SQANTI, is now available to control for the overestimation of novel isoforms in bulk PacBio RNAseq data [66], although the extent of these limitations in single-cell studies has not been assessed yet.

These four requirements (full-length transcript capture; high capture efficiency and sequencing depth; high number of cells sequenced; and low occurrence of errors and artefacts) and how their technological and experimental limitations impact isoform detection in single cells are summarized in Fig. 2. We are confident that these can work as criteria to assist experimental design and provide a framework to assess the success of future isoform studies.

## Expectations meet reality—What has been, and remains to be, done in single-cell isoform studies

**Fig. 3:**
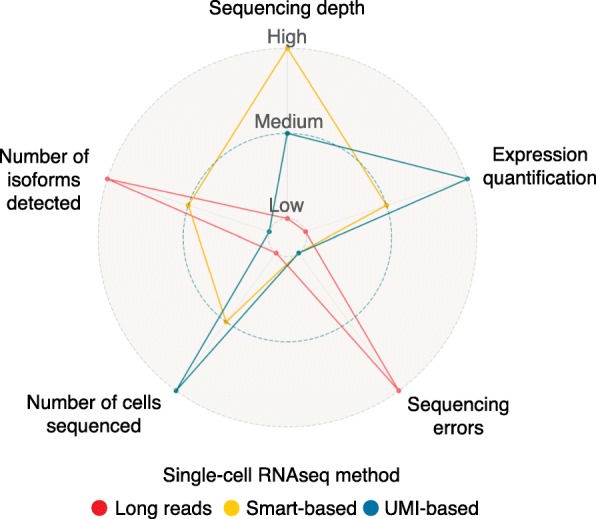
Qualitative performance comparison of the three main single-cell RNAseq methods for isoform detection. From the inside to the outside of the graph, the three *dotted lines* represent ‘low’, ‘medium’ and ‘high’ levels of each characteristic. The most prominent features of long reads (*red*) are high isoform resolution potential but a high occurrence of errors. Smart-based methods (*yellow*) provide high sequencing depth and medium isoform resolution power and number of cells. UMI-based methods (*blue*) can process high numbers of cells with medium to low sequencing depth and accurately quantify isoform expression, although their isoform resolution potential is strongly limited. *UMI* unique molecular identifier

We next discuss how these limitations have been encountered in published studies, and, as a conclusion, present an overall comparison of the performance of the three cited strategies in the single-cell isoform context (Fig. 3).

### Full-length transcript capture and isoform continuity

The full-length requirement inflicts limitations in two ways: first, observation of a limited number of AS events owing to the restricted sequencing length; and, second, less-accurate quantification of isoform expression owing to incompatibility with UMIs.

#### Limitations regarding length

The Smart-seq protocol by Ramsköld and colleagues [39] was the first to improve coverage across the transcript sequence in comparison to prior methods, which possessed strong 3′ bias. Using an RNA dilution of a bulk sample to mimic the amount of RNA in a single eukaryotic cell (~ 10 ng), Ramsköld et al. accomplished a remarkable ~ 40% coverage of the 5′ end. In spite of the coverage improvement, Smart-based protocols (and subsequently most cited short-read studies; Table 1) are limited to quantification of exon inclusion/exclusion.

In the study by Ramsköld et al. [39], an assessment of differential exon inclusion in three cancer cell lines was included in the benchmarking of Smart-seq. In this dataset, 25% of multi-exon genes detected were covered end-to-end, and twice as many differentially spliced exons were detected among the cells when compared with previously published data [11]. Nevertheless, this, together with other studies showing single-cell splicing changes (such as the ones by Marinov et al. [35] and Zhang et al. [40]), was a proof-of-concept isoform study, aiming solely to demonstrate that AS can be studied at the single-cell level.

Regarding mechanisms other than splicing, Karlsson et al. have targeted alternative TSSs [45] using STRT-seq [51], whereas Velten et al. have focused on alternative TTS/poly(A) sites using a novel 3′-targeted method [44]. One advantage of these methods is that they are perfectly compatible with UMIs as they do not span the full transcript. It is interesting to see, however, that Karlsson et al. [45] only obtained a rate of 14% of 5′ end-aligned molecules (obtained by collapsing same-UMI reads) from STRT-seq data, with a 3′-biased coverage distribution ending in a strong 3′ end signal peak—a manifestation of 3′ end bias persistence in short-read sequencing.

Two isoform studies using Oxford Nanopore [46] and PacBio [47] have recently allowed end-to-end characterization of transcript variants for the first time in single cells. Remarkably, Byrne and colleagues [46] have identified an impressive number of alternatively spliced genes (696 using alternative TSS/TTS, and 354 undergoing exon inclusion/exclusion) in B1a cells. Although the level of expression of these isoforms was not quantified, the study shows how SMS can identify larger numbers of AS events than short reads, as expected. In addition, the structures of complex isoforms (in which alternative TSS/TTS and alternative splicing occur simultaneously, as defined in [46]) belonging to 169 genes were identified at an unprecedented level of isoform structure resolution in single cells.

#### Limitations regarding quantification

Initially, the incompatibility of Smart-seq with UMIs was not compensated for by any further assessment or correction of technical variability, including spike-ins, and it cannot be excluded that exon expression estimates published by Ramsköld et al. [39] suffer from technical bias. By contrast, a later study by Shalek and colleagues [36] incorporated validation of results in a dual manner—RNA fluorescence in situ hybridization (RNA-FISH), to compare the isoform ratio differences of two candidates, and a set of additional UMI libraries to exclude the possibility of PCR leading to an overestimation of expression. Validation was successful, indicating that 89 highly expressed isoforms underwent differential exon inclusion across the population.

In a later publication, Zhang and colleagues [40] tested WemIQ (a tool to detect differential exon inclusion in bulk RNA-seq) on the Shalek et al. [36] single-cell dataset. Interestingly, WemIQ removed a great degree of the cell-to-cell heterogeneity from the data, which was attributed to technical bias. As Shalek et al. [36] had reported high levels of heterogeneity in alternative splicing, the WemIQ results raised the question of whether this variability was biological or technical. Simultaneously, this could indicate that bulk RNA-seq tools mistake the higher biological variability in single-cell data for technical noise and points towards the necessity to develop single-cell-specific methods. At any rate, Shalek et al. [36] focused on the bimodality of isoform expression (very high vs very low expression across cells, in a switch manner, synonymous with isoform switches), which can be detected with confidence, even if the results are affected by technical noise.

A first conclusion arising from the above is that, in scenarios where technical bias cannot be properly accounted for, it would be advisable to make a qualitative approximation to isoform variability. In addition, not including UMIs requires other forms of validation, such as RNA-FISH or quantitative PCR (qPCR; as in the Expedition benchmarking [38]), although a limited number of candidates can be validated in this manner. For instance, differential exon inclusion was proven in only two genes in the study by Shalek et al. [36].

An interesting alternative to UMIs was presented in a more recent study by Marinov and colleagues [35]. As a means of estimating noise-contributing factors, and in combination with spike-ins, the authors implemented pool/split controls, produced through pooling several single cells and then splitting the RNA into equal amounts before library preparation. Pooling evens out biological differences between the cells and guarantees that any variability observed will solely be technical, including PCR bias. Differences between controls can then be used to re-estimate cell-to-cell differences. Marinov et al. [35] hereby succeeded to validate isoform switches in 282 multi-exon genes. However, no subsequent studies of isoform diversity at the single-cell level have used pool/split controls.

Regarding quantification and long reads, in contrast to the non-quantitative study by Byrne et al. [46], Karlsson and Linnarsson [47] specifically addressed quantification of isoform expression by optimizing a protocol combining PacBio sequencing with effective resolution of UMIs. Expression estimates in single-cell RNAseq can be used to understand how each transcript might be affected by technical variability or capture issues. In this particular study, poorly expressed isoforms were found to be rarely shared among cells, which provided a means of evaluating sequencing depth limitations.

### Capture efficiency and sequencing depth

Low capture efficiencies yield expression values that will never truly reflect transcript abundances in the cell. In this light, some potentially biologically relevant observations will inevitably be questioned as they could be caused by low capture efficiencies. As an illustration of this, most novel splice sites detected by Marinov et al. [35] are observed only in one cell, which could indicate that these are true isoforms expressed below the detection limits or that they are artefacts. To ascertain whether this is the case, the authors rely on the fact that poorly expressed transcripts are more highly affected by technical noise [60]. As this observation is exclusively true for poorly expressed genes, they conclude that it is less reliable and probably a single-cell technical artefact.

Similarly, in the study by Karlsson et al. [45], a co-expression pattern for TSSs is observed, with good correlation in highly expressed genes, but a weaker relationship as expression levels decrease. Improved capture is proposed as the solution to verify whether TSS expression is also correlated in poorly expressed genes. Interestingly, the cells had been sequenced to an average of 0.5 million reads per cell in the study that generated the data [18], and, although there is probably room to increase the sequencing depth, the low number of reads mapping to TSSs probably would benefit more from higher capture efficiency than from deeper sequencing.

Regarding sequencing depth, numbers of reads per cell of 20 to 40 million have been obtained in single-cell RNAseq isoform studies [36, 38, 39, 44]. This is far above the consensus saturation limit, although no study has addressed how isoform complexity changes with sequencing, and it is unknown whether this is an excess of information for isoform studies, as it seems to be for genes. Nevertheless, it is clear that shallow sequencing can hinder detection of multiple isoforms per gene. This is manifest in the SingleSplice study [42], where the number of detected splice variants and the sequencing depth per cell were shown to follow a linear relationship. Another indicator of unsaturated libraries reported by Welch and colleagues [42] are cells where fewer splice variants than genes were detected. Finally, concerning targeted approaches, higher depth is required to ensure that a sufficient proportion of the reads covers the events of interest. For example, only approximately one-quarter of the total reads per cell in the poly(A) study by Velten and colleagues [44] included polyadenylation sites and were therefore useful for downstream analysis. A similar problem was faced in the investigation of alternative TSSs by Karlsson et al. [45], in which the 3′ bias significantly interfered with the number of reads mapping to the 5′ end.

However, the saturation threshold for long reads is most likely below one million per cell (as reads are not fragmented) and could potentially be estimated as the number of transcripts in the cell lysate. Even so, sequencing depth limitations are exacerbated owing to the trade-off between sequencing depth and number of cells. As an example, Byrne et al. [46] obtained approximately 57,000 to 128,000 reads per cell by multiplexing of four cells on a single MinION flow-cell, and the authors reported difficulties in the identification of low-abundance transcripts and the impossibility of using spike-ins. In the case of Karlsson and Linnarsson’s study [47], a total of six single-cell libraries were pooled and sequenced in a single PacBio-RSII run. In this case, 61% of UMIs were observed only once per transcript, which, owing to the high number of duplicated reads (and hence UMIs) expected following the high levels of amplification required in single-cell RNAseq, the authors concluded was an indicator of sequencing depth limitations. These results suggest that the read-throughput estimates provided by both Oxford Nanopore and PacBio are overestimations, as they significantly differ from that achieved by researchers, and that the current sequencing depth limitation in SMS is a technological one. In spite of this, the isoform detection potential of short versus long reads cannot be faithfully compared solely in terms of sequencing depth—if shallow single-cell sequencing using long-read technologies serves to detect fewer genes, but more isoforms, than using Illumina, a trade-off of quantity for quality might be worth considering in future single-cell isoform studies.

### Number of cells sequenced

Analysing a higher number of cells increases the chances of recurrent detection of novel sites in a bigger cell population, which ultimately increases confidence. Cell throughput is thus recurrently discussed in single-cell isoform studies. Welch and colleagues [42], for instance, observed that few splice variants were detected in more than one cell and highlighted that a higher frequency of detection would have been obtained by sequencing a larger population. Related observations made by Marinov et al. [35]—for instance, that the majority of novel splice sites are present only in a single cell—could have been similarly validated.

In the same way, bimodality and unimodality rates in the study by Song et al. [38] of single-cell neuronal development would acquire more robustness following analysis of a larger population. Even though that study already includes approximately 200 cells (Table 2), there is no current estimate of the minimal number of cells necessary to confidently estimate isoform expression for a given cell type, population or cellular trajectory, but there seems to be room for improvement. For now, deep, Smart-based Illumina sequencing of cell populations is only possible in the range of hundreds of cells [72]. Reassuringly, using Smart-seq and a stringent minimal coverage threshold for splice junctions (i.e. only events covered by at least ten reads were included in subsequent analysis), Song et al. [38] firmly identified 2000–10,000 alternative splicing events in each cell, in spite of cell number limitations.

In the case of long-read technologies, the sequencing depth and budget restrictions pose a limitation upon the number of cells that can be processed. This results in a trade-off between dual reads per cell and cells per experiment. As an estimate, we can consider the MinION experimental design by Byrne et al. [46] (four single cells per flow-cell) as the current maximum capacity of the instrument. Based on these premises, for a 100-cell experiment, approximately 25 MinION flow-cells (which are disposable and can be used in runs of up to approximately 72 h (source http://nanoporetech.com/)) would be necessary. Even though the MinION instrument is cheaper to acquire compared with bench sequencers ($1000 for a starter pack including two flow-cells and a reagent kit), one should note that the cost of the 23 extra flow-cells, plus any additional reagent kits necessary, would rapidly increase the budget to nearly prohibitive costs (source https://store.nanoporetech.com/).

### Sequencing errors and artefacts

Sequencing errors in long-read technologies prevent discrimination of true differential start sites and termination sites from degradation and incomplete reverse transcription artefacts; therefore, TSS and TTS sites have to be defined as nucleotide position ranges (i.e. bins). Consequently, stringent conditions are required for the identification of novel sites, in order to avoid false positives. A particularly conflicting issue is that some of these errors, such as template-switching, arise during reverse transcription, and therefore cannot be identified using UMIs. An alternative is to use spike-ins, which have known sequences, to estimate the probability that such an error occurs and correct it in sequencing data. Using this approach, Karlsson and Linnarsson [47] were capable of attributing an uncertainty of ± 5 bp to the premature termination of reverse transcription (hence variability at the 5′ end), and only considered variation beyond this window as indicating true alternative TSSs. Artefacts can also introduce uncertainty in the identification of exon junctions, which was similarly characterized and corrected using spike-ins in the Karlsson and Linnarsson study.

High error rates also interfere in barcode and UMI resolution. Indeed, Byrne et al. [46] report the impossibility of using UMIs owing to the high error rates of Oxford Nanopore sequencing. In order to be able to resolve them, these authors estimated that UMIs longer than 30 bp would be required, with the subsequent increase in RT and PCR artefacts that such long oligonucleotides would inflict. By contrast, Karlsson and Linnarsson [47] managed to overcome the high occurrence of sequencing errors in PacBio reads thanks to correction of both reads and UMIs by circular consensus sequencing (CCS). It is interesting to keep in mind, concerning barcoding, that PacBio provides the users with a set of 384 barcodes that enables multiplexing of samples, optimized for the technology’s error model (source https://github.com/PacificBiosciences/Bioinformatics-Training/wiki/Barcoding). Oxford Nanopore, in spite of the 2D consensus system, still relies on improvements in sequencing accuracy to incorporate UMIs, and has not developed compatible barcodes for multiplexing. Hence, these need to be designed by the user [46].

Notably, SMS data correction using Illumina sequencing was implemented by Byrne et al. [46] in their application of Oxford Nanopore to single-cell RNA-seq, where support for novel splice junctions by both long and short reads was used as an indicator of high confidence in the true nature of the site. To overcome single-cell limitations, the authors split the cDNA from single cells after library preparation and sequenced it using both Illumina and Oxford Nanopore, although this approach is only feasible when a sufficiently high amount of cDNA has been generated.

Following from our analysis above, none of the three extant methods fulfils the four criteria for successful isoform studies in single cells (Fig. 3). Among them, we have determined that Smart-based methods achieve the best balance, providing high capture efficiency in exchange for reduced statistical power (number of cells analysed). In addition, these methods achieve good numbers of isoforms detected and expression quantification accuracy. However, the latter relies heavily on the computational method of choice, namely on tools to assign reads to the correct transcript isoform, or on other event-based methods, which provide varying approaches to measure splicing changes.

## Computational methods for single-cell isoform studies

**Fig. 4:**
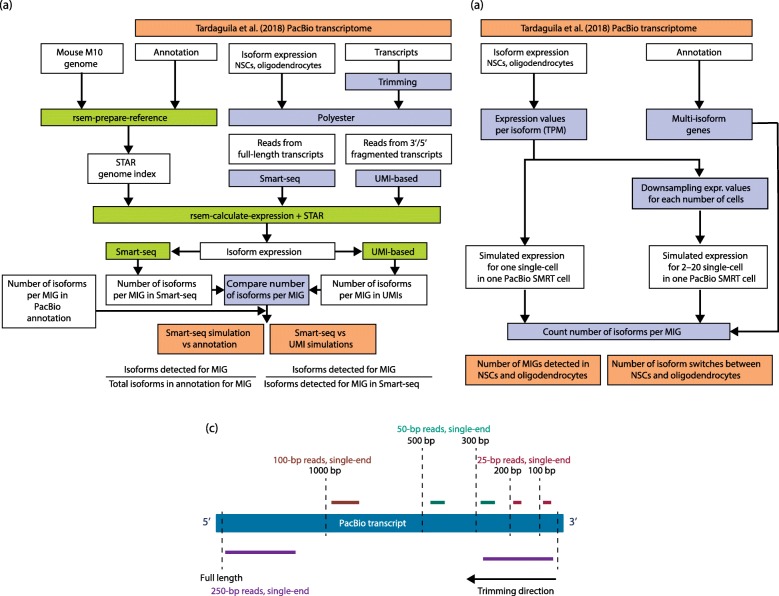
Simulation of short- and long-read workflows and the modelling of a UMI-based library preparation strategy. **a** Short-read simulation workflow. Transcript sequences from the Tardaguila et al. 2018 neural transcriptome [66] were trimmed, and reads simulated from fragments to recreate UMI library preparation limitations in transcript covered length. Full-length reads were also simulated. Reads were aligned to the mouse genome using STAR and isoform expression quantified using RSEM. For UMI simulations, the number of isoforms resolved using Smart-seq reads was used as the 100% reference to calculate the percentage of resolution of MIG. For the Smart-seq simulation, the annotated number of isoforms per gene (in Tardaguila et al. [66]) was used as the 100% reference. **b** Long-read simulation workflow. The Illumina quantification of isoform expression available in Tardaguila et al. [66] was scaled to one million reads (TPM) to recreate a Sequel run of one million long reads, where a single cell is sequenced. Values were downsampled to simulate scenarios where an increasing number of cells (2, 6, 10, 16, 20) are sequenced together in a similar run. The number of reads per cell is therefore gradually decreasing. The number of MIGs in the Tardaguila et al. annotation was compared with the number of MIGs detected in the simulated scenarios. Then, the number of isoform switches detected in the Tardaguila et al. data was compared. **c** Short-read length simulated for each simulation scenario (represented for 3′ UMIs only). PacBio transcript sequences in the Tardaguila et al. dataset [66] were trimmed as described. To ensure that coverage was even when capturing growing lengths of the transcripts in simulated UMI-based protocols, the length of the simulated reads was increased for longer fragments (100 and 200 bp—25 bp reads, 300 and 500 bp—50 bp reads, 1000 bp—100 bp reads, full length—250 bp reads, paired-end). *MIG* multi-isoform gene, *NSC* neural stem cell, *RSEM* RNA-seq by expectation maximization, *TPM* transcripts per million, *UMI* unique molecular identifier

Throughout this review, several computational methods that can be used to study isoform expression at the single cell level have been mentioned. Next, we will provide a more detailed description of the assumptions they rely on and the focus of their results (Fig. 4). Note that we will solely review tools developed for short-read data.

The tools can be divided into three categories: first, methods that detect alternatively spliced genes (i.e. SingleSplice [42]); second, methods that work at the event and exon level (i.e. MISO [41], BRIE [43] and Expedition [38]); and, third, methods that provide a single expression value per transcript isoform (i.e. ‘RNA-seq by expectation maximization’, RSEM [73]) (Table 3).

**Table 3:** Comparative summary of five computational approaches used to study splicing in single-cell RNAseq

	SingleSplice [42]	MISO [41]	BRIE [43]	Expedition [38]	RSEM [73]
Observation level	Gene	Exon	Exon	Exon	Isoform (full transcript)
Measure of expression	Differentially alternatively spliced (yes/no)	PSI	PSI	PSI	Read counts per isoform
Single-cell specific	✓	✗	✓	✓	**?**
Includes interpretation of changes	✓	✗	✗	✓	✗

*PSI* percentage spliced-in, *RSEM* RNA-seq by expectation maximization

To deal with the limitations of single-cell RNAseq, SingleSplice introduces the novel concept of ‘alternative splicing modules’ (ASMs, referred to as ‘splice variants’ in the previous section for clarity). The power of ASM lies in the fact that isoforms that differ in junctions near the 5′ end are automatically grouped together under the same ASM and assigned a combined expression value. In this manner, SingleSplice does not need to discriminate all isoforms, only as many as possible given the lack of 5′ coverage and the limitations intrinsic to assigning short reads to transcripts. Once the tool has identified all ASMs that belong to each gene, it looks for genes showing cell-to-cell changes in ASM expression. This can be considered a ‘zooming out’ approach, focusing on identifying genes that are alternatively spliced in a given biological context, instead of identifying particular isoform changes.

Although MISO was developed to detect alternative splicing in bulk RNAseq, it has been applied to early single-cell studies that incorporated isoform diversity [36, 39]. Instead of reconstructing full transcripts from short reads, the tool uses reads aligned to splice junctions and a mixture model to estimate percentage spliced-in (PSI) values for alternatively spliced exons. PSI is defined as the fraction of mRNAs that represent isoforms where the exon is included. This value depends on the number of reads aligning to the exon, the flanking constitutive exons, their junction and the bodies of other constitutive exons, which contain information on the abundance of both the inclusion and exclusion isoform. To incorporate the latter, inference of PSI for each exon is treated as a Bayesian problem, and confidence intervals are used to evaluate the reliability of the PSI estimates.

BRIE and Expedition build on the same premises as MISO and assess expression at the exon level. Nevertheless, these tools use new strategies to face challenges specific to single-cell data. In particular, they differ in the way they quantify events (1) and in their approach to evaluate splicing across cells (2).

Regarding (1), in the case of BRIE, isoforms are not defined as full transcripts, but as exclusion/inclusion isoforms for each alternatively spliced exon. For exons where read count is high, a mixture model approach similar to that of MISO is used. In addition, however, informative priors learned from the data are used in a Bayesian regression model in order to improve sensitivity and obtain accurate estimates where reads are sparse. This feature can also be used for drop-out imputation. By contrast, Expedition exclusively uses junction-spanning reads for quantification, but is rather conservative to only quantify sufficiently covered, reliable events (> 10 reads), as opposed to the greedier approach of BRIE.

As for feature (2), the modelling strategy used by BRIE allows good quantification but is limited to relative inclusion rates (PSI defined as in MISO). In addition, once PSI is estimated for all events in the cells, pairwise comparisons are used to detect differences between cells. This is both computationally costly and impractical, particularly when high numbers of cells are analysed. For Expedition, the authors instead define an absolute PSI measure (a 0 to 1 value that indicates the percentage of transcripts per cell that include a given exon) used to measure exon usage at the single-cell level. The tool then classifies events into ‘modalities’ according to their distribution of PSI scores in the overall cell population. The classification used by Expedition is useful to understand global trends for each event, as well as to assess changes in these trends across cell types or conditions. Consequently, Expedition yields more easily interpretable results than the pairwise comparison strategy of BRIE.

Finally, it is currently possible to use the bulk-designed tool RSEM [73] specifying a single-cell parameter option, a feature added in a 2015 release. When selected, RSEM uses a sparse prior for its Expectation Maximization algorithm in order to better account for the characteristics of single-cell RNAseq data when assigning reads to transcripts. This provides a single expression value per annotated isoform. However, this feature has not been benchmarked using single-cell data, and therefore it is currently unknown whether the expression estimates provided are sufficiently accurate.

Choosing one tool over another depends mostly on the aim of the study. SingleSplice provides a general overview of the consistency of splicing for all multi-isoform genes in a given population, which can be selected when event or isoform-level resolution is not required. For information on splicing changes involving particular events that might be interesting in a given population of cells, Expedition is recommended. Finally, RSEM is the only available tool that provides a single expression value per transcript isoform, although its performance on single-cell data has not been tested. Note that, to make the most of isoform-level assessment, a comprehensive annotation of the full-length isoforms in the sample is recommended.

## What are the theoretical limits of current technologies for single-cell isoform studies?

We have described technological limitations and discussed results obtained in the analysis of isoform expression at the single-cell level. One immediate question is to what extent characterization of isoform diversity in single cells is actually feasible given the current state of the art. In order to gain insight into this issue, we ran a simple simulation experiment where single-cell transcriptomics data for the different technological approaches were emulated. As a reference, we used data from a recently published bulk-RNAseq study, which sequenced the RNA from mouse neural progenitors (NPCs) and oligodendrocytes using the long-read PacBio platform [66] and quantified isoform expression using Illumina short reads. This dataset comprises approximately 0.6 million PacBio and 60 million Illumina reads per sample, resulting in approximately 13,000 full-length transcripts belonging to approximately 7000 genes, 45% of them multi-isoform genes (MIGs; described in the annotation available elsewhere [66]). Basing this simulation on a PacBio dataset allows the consideration of transcripts expressed specifically in these cell types. Note that by basing our simulation on RNAseq data we assume similar isoform diversity at the cell and bulk levels, which is unlikely, but sets a theoretical maximum for single-cell transcriptome complexity. Based upon this, other factors such as sequencing depth and library construction strategy were then assessed, as described below.

### UMI versus Smart-based methods—Partial sequencing of transcripts limits isoform resolution

**Fig. 5:**
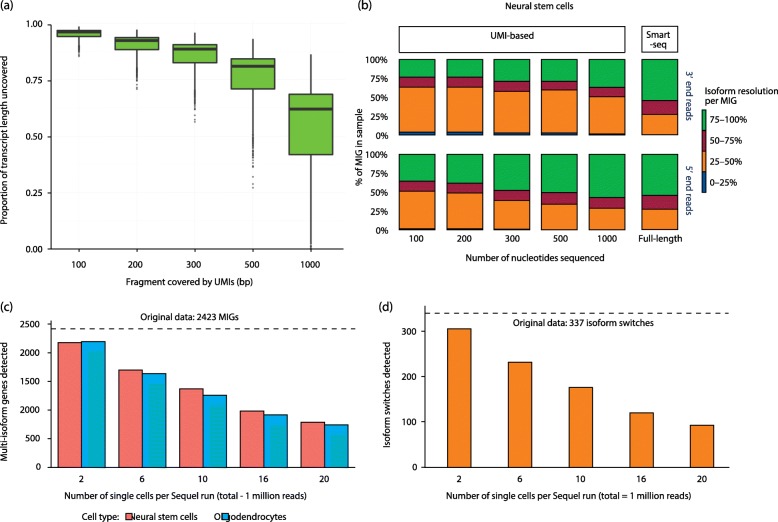
Simulation results. **a** Short-read simulations—proportion of transcript length left uncovered as longer fragments are simulated in a UMI-based library preparation scenario. Short fragments (100–200 bp) leave most of the transcript uncovered by the reads (> 0.75 proportion), while the simulation of longer (> 300 bp) fragments affects transcripts differently depending on their length, hence the growing distributions in the boxplot. **b** Short-read simulations—multi-isoform genes (MIGs) detected in each 3′ and 5′ end as well as in the Smart-seq simulation are classified in four intervals, according to their individual percentage of resolution. Results shown for neural stem cells (NSCs) only. Intervals gather MIGs for which 0–25, 25–50, 50–75 and 75–100% of their isoforms are resolved. The 3′ end and 5′ end labels only refer to unique molecular identifier (UMI) simulations. Note that Smart-seq data have been plotted twice, in both the 3′ end and 5′ end bar-graph rows, for completeness and to ease visual comparison. **c** Long-read simulations—the number of genes for multi-isoform genes detected as sequencing depth per cell is progressively lost. The *dashed line* indicates the number of multi-isoform genes present in the original neural cell transcriptome. A decrease in depth per cell decreases the number of genes for which more than one isoform can be observed. **d** Long-read simulations—the number of isoform switches detected between neural stem cells and oligodendrocytes in a similar scenario, assuming half of the cells belong to each cell type (i.e. two cells equate to one oligodendrocyte and one NSC). A decrease in sequencing depth per cell not only prevents detection of isoform ratio expression changes (which constitute the majority differences in isoform expression), but also reduces the number of isoform switches that can be observed. The *dashed line* indicates the number of NSCs versus oligodendrocyte isoform switches detected in the original transcript expression data

Related to the effect of library preparation upon transcript coverage using short-read sequencing, we simulated how the partial 3′ (and 5′) end sequencing intrinsic to UMI-based methods limits the number of isoforms that can be detected (for detailed workflow, see Fig. 4a). The polyester R package [74] was used to simulate reads from a growing length of the 3′ and 5′ ends of the neural full-length PacBio transcripts. To simulate this, we took advantage of the fact that polyester requires transcript sequences as a template to generate short reads and of the number of reads to generate per transcript. We trimmed the sequences of transcripts in the PacBio transcriptome to lengths of 100, 200, 300, 500 and 1000 bp, starting from the 3′ and 5′ ends and independent of the length of each transcript, and used them as the input template transcripts to polyester. In this scenario, polyester generated short reads from limited portions of the transcripts. In this manner, we recreated a range of simulated UMI-based library preparation results. Note that our simulation does not capture a real UMI library scenario, where covered lengths vary from transcript to transcript and duplicated reads are collapsed. However, as longer fragments are sequenced, an increasing number of AS events is expected to be captured, which is sufficient to illustrate the limitations in covered transcript length extant in both 3′ and 5′ UMI-based methods (Fig. 5a). In parallel, a second set of short reads was simulated spanning the entire transcript sequence, recreating a Smart-based library preparation strategy (Fig. 4c).

For all simulated samples, a total of one million reads were generated. To achieve this, the expression values obtained for both NPCs and oligodendrocytes using Illumina reads in the neural cell-type study [66] were scaled to one million (transcripts per million (TPM)) and used as input to polyester. This ensures a realistic range of expression values, maintained across simulated samples. As the total reads and reads per transcript are constant, even coverage was controlled by gradually increasing read length with the fragment lengths (Fig. 4c). In addition, single-end reads were simulated for shorter fragments, whereas paired-end reads were generated for a full-length transcriptome scenario. Isoform expression was calculated using RSEM [73] with STAR [75] in order to obtain a single expression value per transcript isoform. Then, the number of isoforms detected per multi-isoform gene (MIG) simulation was calculated and compared (Fig. 5b).

Figure 5b shows that, for a 3′ end UMI library where reads are generated from the first 100 bp of the transcript, only 25% of the MIGs would be near to fully resolved (green bar section, MIG where > 75% of its isoforms are discriminated). This group comprises MIGs where events decisive for isoform discrimination occur near the 3′ end. However, for the majority of genes, 25 to 50% of the expressed isoforms would be discriminated, meaning that most of the splicing variations occur beyond the last 100 bp and are being missed. Covering a longer fragment of the transcript molecules would only improve results marginally, according to the minimal increase in the numbers of MIGs resolved at > 75% resolution in the 200 to 1000 bp 3′ UMI simulations. Our simulations suggest that the percentage of MIGs falling into this category is slightly higher if UMIs are 5′ end located (Fig. 5b, second row of bars).

Interestingly, methods based on the Smart-seq2 protocol would resolve well ~ 50% of the MIGs at the simulated one million reads, when arguably a higher percentage would be expected. This can be attributed to a failure to capture poorly expressed transcripts in the original data and/or limitations of the RSEM algorithm when resolving highly similar isoforms using short reads. Arguably, then, a single-cell Smart-seq experiment will always suffer the pitfalls intrinsic to short-read isoform reconstruction, which should be accounted for when interpreting results. Finally, it should be noted that very similar trends were observed when simulating neural stem cells (NSCs; Fig. 5b) and oligodendrocyte samples (data not shown).

### Long reads—Illustrating the trade-off between cell number and sequencing depth

The major limitation of long-read technologies for single-cell sequencing is the impossibility of achieving deep sequencing at an affordable cost. To understand the implications for isoform detection, we simulated a scenario where an increasing number of cells were sequenced by one PacBio Sequel run of a theoretical one million full-length reads. In such a situation, where maximum depth is fixed, the number of reads per cell decreases as the cell number sequenced per run increases. Next, we calculated the number of genes for which more than one isoform can be detected and the number of isoform switches that can be observed between NSCs and oligodendrocytes in each simulated scenario.

To simulate this, we downsampled the bulk transcript expression results obtained in neural cell types [66], assuming equal distribution of the reads among cells. Using bulk data allows one to work with a theoretical maximum of transcript detection, but presumably the drop-outs in a real single-cell scenario would play an important role. The results discussed below should therefore be interpreted as upper-bound estimates. Single-cell transcript expression results were generated for 2, 6, 10, 16 and 20 cells, and the simulation workflow is detailed in Fig. 4b.

Not surprisingly, we found that the number of genes for which more than one isoform is detected decreases with sequencing depth (Fig. 5c). This is inevitable as shallow sequencing will more easily capture highly expressed transcripts but will miss alternative isoforms. Single-molecule technologies might, however, still be able to capture differences in isoform expression when they imply drastic changes in expression—that is, isoform switches. We evaluated this by computing the average number of isoform switches between the NSC and oligodendrocyte simulated single-cell transcriptomes (Fig. 5d). We found that, for a total of 2423 multi-isoform genes detected in the original data [66], only 337 (~ 14%) undergo isoform switches. This means that, as expected, most isoform changes constitute expression ratio variations. Most of them (305) are detected in the best-case scenario of our simulation (one NSC and one oligodendrocyte cell), although the number decreases as the number of reads per cell decreases. For example, in a 20-cell experiment, only about one-third of these changes can be detected, according to our simulation. Therefore, we can anticipate that favouring cell number over sequencing depth will lead to missing the majority of isoform expression changes in a cell population.

## Attributing biological significance to isoforms at the single-cell level

While the functional role of alternative isoform expression is the subject of intensive research and discussion, specifically, the aim of single-cell studies is to evaluate isoform prevalence in cell populations, either for subpopulation characterization (#1), to assess the importance of isoform expression changes in dynamic processes (#2) or to investigate its stochastic nature (#3).

A remarkable example of (1) is how, as opposed to population-level estimates of isoform abundance—which indicate co-expression of the different splice variants of a gene—single-cell RNAseq has shown that not all cells express all isoforms, but predominantly show either exclusion or inclusion of the exon, revealing a bimodal, switch-like pattern for splicing across immune cells [36]. A later study by Marinov et al. [35] consistently found bimodality in isoform expression in a related immune cell type. In it, stochastic gene expression bursts are proposed as an explanation for isoform dominance in cell subpopulations over space and time, a hypothesis that aims towards answering question #3 proposed above. These findings, however, conflict with evidence that cell-specific isoform co-expression is tightly regulated in the nervous system, where it is essential to the formation of synapses, neuron self-recognition and gene expression homeostasis [37]. Similarly, observations made by Karlsson et al. [45] support the idea that TSSs are mostly co-expressed in the mouse brain, and that, therefore, TSS usage is a co-regulated process and not a stochastic one. Moreover, recent research on neural differentiation has found that the majority of isoforms were expressed in accordance with a unimodal pattern—that is, that the dominating isoform tended to be the same in the entire population of cells [38], in line with Shalek et al. [36] and Marinov et al. [35]. Concerning differentiation dynamics (#2), however, Song et al. [38] observed that this was not a static behaviour, but that 20% of alternative splicing events shared among the population changed during differentiation. Changes were mostly from a unimodal pattern, where all cells presented either the inclusion or the exclusion isoform, to a bimodal pattern where two subpopulations, each one predominantly expressing one of these variants, could be identified. This seems to indicate that isoform switches (i.e. switching of the dominant isoform) could be a useful tool to examine cell subpopulation differences, and has in fact been used by Song et al. [38] to separate distinct subpopulations appearing during the neural differentiation trajectory. However, these studies have only been conducted at the event level, and therefore it has not been investigated whether transcript isoform expression in single cells follows similar trends. Therefore, how an isoform-level analysis would contribute to answer the three questions we propose is still unknown.

Regarding studies using SMS, which have the potential to enable full-length isoform studies, sequencing depth and cell number limitations have for now prevented conclusions tackling these questions. However, the two studies published to date [46, 47] are groundbreaking in terms of demonstrating that it is possible to use long-read technologies in single-cell RNAseq. We anticipate that future improvements by PacBio and Oxford Nanopore will allow more biologically oriented studies to refine and complete the hypotheses developed from short-read studies on the single-cell biology of isoform expression.

Another aspect that is relevant for understanding the biological role of isoforms is how changes in their expression are connected to other layers of gene regulation. To this end, methods to generate other types of single-cell data, such as single-cell epigenomics, are beginning to appear. These kinds of technologies are bound to be combined with transcriptomics into single-cell multi-omics and data-integration approaches, as has been reviewed recently [76]. However, this approach to single-cell biology is still in its infancy, and there are few examples of these types of studies (see [77–81] for single-cell multi-omics, and Lake et al. [82] for a data-integration approach). In addition, none of them, to the best of our knowledge, includes alternative splicing, TSS or polyadenylation information within their transcriptome data, only gene expression. In order to build complex models that include these isoform-associated forms of regulation, multi-omics technologies must use Smart-based methods (or, in the future, long reads) to obtain transcriptomics data. Interestingly, two recently developed single-cell multi-omics methods, scM&T-seq [81] (methylome and transcriptome) and scNMT-seq [78] (chromatin accessibility, methylome and transcriptome) use Smart-seq2 to profile the transcriptomes of cells. Even though these data have not been analysed at the isoform level, these protocols are potentially useful to understand how the expression of alternative isoforms is coupled to other gene-regulation mechanisms.

Alternative isoforms are the result of alternative splicing, as well as changes in TSS and polyadenylation site usage. Single-cell RNAseq has demonstrated great potential to characterize the diversity of isoforms that exist in a single cell. The main challenges facing the field are conferring biological entity to this diversity by answering the three questions mentioned: determining the importance of isoform expression in defining the identity of cell types (#1), the biological role of the expression changes observed in dynamic processes (#2) and the degree of stochasticity of the mechanisms by which they occur (#3).

## Concluding remarks and future perspectives

We have described how current limitations of isoform studies using single-cell RNAseq impact investigations published within the field, together with the main considerations that should be taken into account before producing isoform data. Given the novelty of this application, we believe that this review will be useful to inform experimental design, as we have both enumerated present experimental concerns and provided guidance as to how to maximize isoform detection. Even so, most limitations of single-cell RNAseq cannot be solved through experimental design as they are fundamentally technological.

Short-read sequencing is mainly limited by library preparation protocols. Although current Smart-based protocols are capable of detecting transcripts from up to ~ 20,000 genes [61], this is not always sufficient to capture rare isoforms. A second key issue is the number of cells that can be sequenced at high depth, at present limited by the cost per cell of the Smart-seq2 protocol, which is not able to compete with low-coverage, high-throughput protocols such as Drop-seq. Interestingly, cost reduction has been reported when producing an in-house transposase [61, 83], although in this case Smart-seq2 still qualifies as more expensive than most of the other protocols and thus needs optimization. Last, but not least, current full-length protocols do not enable inclusion of UMIs for PCR bias detection and, furthermore, are not completely efficient in capturing the 5′ ends of transcripts.

To compensate for the incompatibility with UMIs, new experimental controls could be developed to refine quantification. In fact, the necessity to develop a set of spike-in RNAs that is more adequate for single-cell RNAseq, given the biases that widely used ERCC materials suffer from, has recently been pointed out [84, 85]. Alternatively, new computational approaches where true transcript abundances are estimated without the necessity to use UMIs—or spike-ins—are beginning to appear. Such is the case of Census [86], a tool that, based on the assumption of linear amplification, estimates relative transcript counts initially present in the cell lysate. Even though linear amplification constitutes a bold assumption, authors report that Census performs more accurately than normalized read counts when UMIs and spike-ins are incompatible with the experimental design. Census opens an interesting path towards estimation strategies along these lines that could aid isoform studies in the future.

Also on the computational side, a robust, manageable and easily interpretable estimation of exon inclusion is required for short-read studies. Several strategies have recently been developed for single-cell data [38, 42, 43]. The necessity to adapt estimates to the peculiarities of single-cell RNAseq data has been based upon three considerations: first, high levels of technical noise (addressed by Welch et al. in SingleSplice [42]); second, high processing requirements for single-cells (addressed by Song et al. in Expedition [38]); and, third, misquantification of poorly expressed isoforms caused by lack of coverage in low-expression ranges (addressed by Huang et al. in BRIE [43]). The cited studies use diverse strategies to overcome a common problem, which results in different isoform expression metrics. Future research, by contrast, should aim to bring the field closer to a standard way of measuring isoform expression. Standardization would not only save time comparing the performance of the different tools, but also make single-cell-isoform studies significantly more comparable. More importantly, however, the field lacks a single-cell-specific tool that provides one expression value per transcript isoform, as opposed to the assessment of single splicing events. Isoform-level expression estimation integrates the whole combinatorial diversity of splicing, TSS and poly(A) events, and will ultimately enable complete, system-level assessment of the role of splicing in generating functional diversity and its interplay with other layers of gene regulation. Therefore, we recommend evaluating transcript isoform expression in this manner, currently only possible using RSEM [73].

Concerning computational approaches, long-read sequencing lags behind in comparison with strategies using short-read data. To illustrate, the first computational workflow, *Mandalorion* [46] was developed and published very recently by Byrne and colleagues and focuses on identification of isoforms without expression quantification (furthermore, the pipeline has not been scaled and released as a compact tool). This application of SMS is in a very preliminary phase, where experimental design and technological limitations hinder high-throughput and high-quality data production. Two issues currently require improvement—flow-cell capacity and sequencing accuracy. Flow-cell capacity refers to achieving higher depth per cell, through both the development of zero mode waveguides (ZMWs, PacBio) and nanopores (Oxford Nanopore) that resist pre-run inactivation, as well as an increase in cost-effectiveness, oriented towards lowering costs per cell, per run and per flow cell. In this context, the much expected release of the throughput-enhanced Sequel (PacBio), GridION and PromethION (Oxford Nanopore) sequencers is a promising next step.

In the light of the current limitations, alternative approaches can be proposed. The first one is the possibility for combining long-read sequencing with prior selection of homogeneous cell subpopulations. Specifically, a large cell population could be screened and sorted according to biological properties of interest (i.e. by flow cytometry), generating pools of a few thousand cells. Pooling cells would improve read depth and capture efficiency to levels near bulk RNAseq, while subpopulation selection would facilitate getting beyond the bulk population level. Nevertheless, subpopulation homogeneity can be assumed only to a certain extent, and selection requires a considerable amount of previous knowledge. In addition, this strategy excludes cell-level pattern observation. Second, targeted sequencing using PacBio or Oxford Nanopore could be used to characterize isoform sets belonging to genes of interest—that is, genes that are known to have isoform diversity in the biological context under study, which could be detected via bulk RNAseq. As a result, only a fraction of the diversity in the single-cell isoform landscape would be detected, but the limited read depth and cost restrictions of both SMS technologies would be overcome.

In conclusion, no currently available single-cell RNAseq strategy (nor alternative one) performs optimally when isoforms are the aim of the study. In this context, future advances will rely strongly on the ability of researchers to design alternative experimental solutions to complement the gap left by single-cell sequencing and, more importantly, on technological improvements in both library preparation and sequencing protocols. Reassuringly, the increasing number of studies using single-cell RNAseq will certainly make such progress a reality sooner than later. Hence, the ultimate challenge for single-cell isoform studies will be to rationalize the biological significance of isoforms—that is, whether such high diversity truly constitutes an extra layer of regulation in the cell or, on the contrary, is solely a manifestation of the stochasticity that governs biological systems.

## Abbreviations

AS: Alternative splicing
ERCC: External RNA Control Consortium
MIG: Multi-isoform gene
NSC: Neural stem cell
PSI: Percentage spliced-in
RT: Reverse transcription
SMS: Single-molecule sequencing
TSS: Transcription start site
TTS: Transcription termination site
UMI: Unique molecular identifier
UTR: Untranslated region
